# Referral of patients with chronic kidney disease: inconsistencies between guidelines and real-life practice, more questions than answers

**DOI:** 10.1007/s40620-022-01249-5

**Published:** 2022-02-14

**Authors:** Massimo Torreggiani

**Affiliations:** Centre Hospitalier Le Mans, 192 Avenue Rubillard, 72037 Le Mans, France

Chronic kidney disease (CKD) affects about 10–15% of the global population, according to different estimates, and is a leading cause of morbidity and mortality worldwide [1]. In recent years, the focus has been on the growing incidence of CKD, leading to reflection on which strategies would best enable us to deal with an ever-increasing demand for specialist care.

In this issue of the Journal, Schultz and colleagues report on the discrepancy between nephrology referral criteria suggested by different guidelines and real-life practice [2]. Their study shows that in nephrology referral the Canadian guidelines are those which most closely mirror what happens in clinical practice. They found that only 42% of patients referred match the indications of the Kidney Disease Improving Global Outcomes (KDIGO) guidelines and slightly more than half of patients are referred in accordance with the French guidelines. Moreover, while according to current guidelines most of the patients referred did not need to be seen by a specialist, only 10% of the total sample was directly discharged by the nephrologist after consultation and had no further follow-up. This last point raises a number of questions that warrant reflection.

While one major issue, the evident need to align guidelines, is a topic that is beyond the scope of this editorial, considering several of the other points that emerge from the study could stimulate constructive debate.

First of all is the question concerning what role guidelines play in regard to nephrology referral. Let us consider the KDIGO guidelines on CKD, now a decade old, according to which referral to a nephrologist is advised when the glomerular filtration rate (GFR) has fallen below 30 ml/min/1.72 m^2^ or in the presence of significant proteinuria, sustained unexplained microhematuria, progression of CKD, potassium alterations, a family history of kidney disease, recurrent nephrolithiasis, with a quality of evidence graded “moderate” [3]. Schultz’s work shows that most patients were referred on the basis of GFR rather than degree of proteinuria [2]. In this context, the cut-off point at 30 ml/min/1.72 m^2^ becomes a two-faced Janus: it is a clear indication that a patient must be referred to a nephrologist, but it is also an incongruent threshold in the attempt to halt, or even only slow disease progression, at least according to the recent KDOQI guidelines on nutritional management in kidney diseases [4]. The first alternative represents the premise upon which the Haute Autorité de Santé (HAS—the French national healthcare system) has moved to implement a new reimbursement bundle for integrated care of CKD in order to ameliorate nephrology care in France.

Taking advantage of this new reimbursement system, our group analyzed the need for nephrology care and proposed a simple way to estimate it in keeping with the local situation [5]. While the nephrology workforce we have is insufficient for coping with the number of patients in CKD stage 3 needing specialized nephrology care, based on the prevalence reported in the literature, we conservatively estimated that about 2000 CKD stage-3 patients per million population have progressive diseases but are not seen by a nephrologist. In this estimate we did not consider the indications of the guidelines analyzed by Schultz and co-workers, but followed the philosophy of the most recent Kidney Disease Outcomes Quality Initiative (KDOQI) guidelines on dietary management of CKD [4].

These guidelines hold that all CKD patients, regardless of stage, should undergo regular assessment of nutritional status, and that starting in stage 3, in the absence of contraindications and without age restrictions, they should be managed with a low-protein diet to slow the progression of kidney disease and improve uremic symptoms and metabolic control; this intervention (rated A1, the highest evidence level) is associated with better survival and lower mortality [4]. The intervention should be applied “under close clinical supervision”, but it seems unlikely that primary care physicians would embark on such a task requiring specific training and a multidisciplinary approach (nephrologists, trained dietitians, etc.). Moreover, considering France where Schultz’s study was conducted, the lack of primary care physicians is a longstanding issue, adding to the complexity of the matter.

Furthermore, how can we be sure that earlier CKD stages should be denied referral to a specialist?

CKD has a heterogeneous progression towards end-stage kidney disease (ESKD) which depends on primary disease, concomitant risk factors and comorbidities, sex and age [6]. Moreover, the progression of CKD is often non-linear, thus complicating its evaluation [7].

Considering the relationship between renal function and histological correlates, due to the large kidney function reserve, a rise in serum creatinine only becomes evident when more than 50% of the nephron mass has been lost. Thus, if we have a chance to modify the natural course of CKD, it is most likely that this will be in its initial stages, with targeted interventions when kidney tissue is still relatively preserved.

Finally, there is a huge difference in clinical practice between high- and low-healthcare resource countries, and universal guidelines developed in high-income settings may not be consistently applied worldwide but rather adapted to the local situation. A study from the United Kingdom showed that implementing eGFR for specialist referral led to a 265% increase in referrals in the first months since the program started but, later, it decreased to a steady rate of 40%, questioning the role of overwhelmed nephrology units [8]. Geographical epidemiological differences account for different needs: the increase in life expectancy in the western world accounts for the increase in later CKD stages while, in developing countries where there is a substantial lack of resources, earlier stages predominate [9].

For the reasons set forth above, ideally all patients with kidney alterations should be seen by a specialist at the first sign of disease, at least in a first phase, to determine whether to continue nephrology follow-up. Difficulties in predicting who will progress and who will not can explain the low rate of discharge of patients observed in Schultz’s study and can be the reason for the retention of patients in nephrology clinics: a prudent attitude of common sense in the light of our patients’ interest. The need to find a compromise between clinical needs and medical desertification, within healthcare systems already stressed by increasing pressure, should not influence guideline recommendations if we want to prevent ESKD.

In conclusion, concerning referral of CKD patients to a nephrologist, rather than focusing on why physicians deviate from guidelines, should we not reconsider the guidelines, so that they do more to take patients’ needs into account?
